# Comparing and Contrasting the Impacts of Macro-Level Factors on Crash Duration and Frequency

**DOI:** 10.3390/ijerph19095726

**Published:** 2022-05-08

**Authors:** Sai Chand, Zhuolin Li, Abdulmajeed Alsultan, Vinayak V. Dixit

**Affiliations:** 1 Research Centre for Integrated Transport Innovation (rCITI), School of Civil and Environmental Engineering, University of New South Wales, Sydney, NSW 2052, Australia; zhuolin.li11@student.unsw.edu.au (Z.L.); v.dixit@unsw.edu.au (V.V.D.); 2 Department of Civil Engineering, Prince Sattam Bin Abdulaziz University, Al-Kharj 16278, Saudi Arabia

**Keywords:** crash duration, crash frequency, hazard-based, Negative Binomial, latent class

## Abstract

Road traffic crashes cause social, economic, physical and emotional losses. They also reduce operating speed and road capacity and increase delays, unreliability, and productivity losses. Previous crash duration research has concentrated on individual crashes, with the contributing elements extracted directly from the incident description and records. As a result, the explanatory variables were more regional, and the effects of broader macro-level factors were not investigated. This is in contrast to crash frequency studies, which normally collect explanatory factors at a macro-level. This study explores the impact of various factors and the consistency of their effects on vehicle crash duration and frequency at a macro-level. Along with the demographic, vehicle utilisation, environmental, and responder variables, street network features such as connectedness, density, and hierarchy were added as covariates. The dataset contains over 95,000 vehicle crash records over 4.5 years in Greater Sydney, Australia. Following a dimension reduction of independent variables, a hazard-based model was estimated for crash duration, and a Negative Binomial model was estimated for frequency. Unobserved heterogeneity was accounted for by latent class models for both duration and frequency. Income, driver experience and exposure are considered to have both positive and negative impacts on duration. Crash duration is shorter in regions with a dense road network, but crash frequency is higher. Highly connected networks, on the other hand, are associated with longer length but lower frequency.

## 1. Introduction

Traffic incidents such as vehicle breakdowns, accidents, and hazards have a significant impact on traffic performance, resulting in traffic congestion, unreliability, and pollution [1]. Crashes can also have profound emotional, physical, economic and social implications. Researchers have used a variety of variables to assess safety, including crash frequency, rate, severity, and duration. However, the number of research articles on crash frequency far outnumbers other metrics, likely to be because of the relative ease of obtaining frequency data [2].

Vehicle crashes, as an example of unplanned incidents, not only affect typical traffic conditions but have also been regarded as the eighth leading cause of death in all age groups worldwide [3]. Due to their high injury and fatality rate, crashes have always been a critical component of safety analysis. Although human factors are considered to be the main contributor to crashes, those caused by other variables (e.g., roadway characteristics, environment) are relatively more predictable and preventable. After the occurrence of the crash, the assistance of responders such as police, roadside assistance, medical and rescue departments is necessary in most cases. Generally, the longer responders take to reach the scene, the more severe the accident tends to be and the longer it lasts. Therefore, it is essential to have a comprehensive understanding of the nature and pattern of crash occurrence and clearance, and to implement appropriate incident management strategies. With this intention, identifying the key variables that affect such events from the past crash records is crucial.

Crash analysis is performed at the macro and micro levels. In the macro-models, the area of interest is divided into traffic analysis zones (TAZ), census wards, statistical areas, etc. The impact of macro-level covariates, such as socioeconomic, demographic, environmental, infrastructure, and traffic patterns, are modelled to provide countermeasures from a planning standpoint [4]. On the other hand, micro models evaluate crashes on a highway segment or intersection. Here, the variables, such as geometric design, signage, sight distance requirements, time of day, etc., are considered from the engineering and operational standpoint. 

Previous studies on crash duration usually extracted potential variables directly from the crash records. These variables comprise crash characteristics, environmental and temporal features, traffic flow, and the roadway geometry where the incident has taken place. Consequently, the exogenous variables are localised due to the selection of highway segments as the study scope [5,6]. The effects caused by macro-level factors have not been thoroughly investigated in past studies, contrary to research on crash frequency. Conducting a zonal level analysis of crash duration can help to better understand how factors that significantly contribute to crash occurrence affect the post-incident response process, thereby helping to minimise the risk of secondary incidents.

Different statistical methods have been used and compared against one another in past research, but most studies only focus on a single safety metric. Thus, the focus has been on the predictive power of different statistical models. A few studies jointly modelled some metrics, notably crash frequency and severity [7,8,9]. However, studies analysing the influence of the same explanatory variables (from the same study area and, thus, dataset) on crash frequency and duration cannot be found in the literature. This research aims to compare and contrast the impacts of different explanatory variables on crash frequency and duration. Specifically, this study looks at determining: (a) whether a macro-level variable that significantly impacts crash frequency has any impact on duration or vice-versa, and (b) if the variable is significant in both cases, whether the direction of the impact is the same, or not.

The study also fills the gap in past studies that have not sufficiently examined the effect of street network patterns on highway safety. Over the last four decades, there has been a declining trend in the number of people killed or injured in car accidents in more than 30 countries. This could be explained by increasing recognition of traffic safety and improved vehicle safety performance [10]. Additionally, the enhancements in general road network structure and design could also be one of the reasons. However, this impact brought about by the network structure is underestimated by previous studies and practice. Only a few studies have analysed the safety effects of road network structure metrics, such as density and connectivity [11,12]. Even so, these studies focus on either crash frequency or crash rate, with no attention to other metrics, e.g., crash duration or severity. 

The rest of the paper is organised as follows. First, a review of methods employed for crash frequency and duration is provided. It is followed by an overview of the various statistical models utilised in the paper, then by a description of the study area, crash data and potential influencing factors. The results of the modelling are then presented, together with information on the effects of key parameters on accident duration and frequency. The last section summarises the most important findings and suggests future study directions.

## 2. Literature Review

### 2.1. Crash Frequency

Crash count data modelling is an important branch of traffic safety analysis. In macro-level analysis, researchers usually divide the area of interest into common units such as traffic analysis zones (TAZ) [13,14], and census wards [15], or other units which are based on statistical level [16], uniform size grid [17], and cities [18]. A variety of covariates including socio-economic and demographic, environmental and temporal, infrastructure and road network (e.g., road density and length, network connectivity and circuity), vehicle characteristics, and driver behaviour and licensing, traffic pattern variables (e.g., average driving speed and traffic volume) are typically considered. Among these, socio-economic and demographic features consist of population densities, age-group distributions, per-capita income, employment rate, GDPs, number of hospitals and medical services, crime rates, suicide rates, and police enforcement. Temporal and environmental covariates can further be classified as average temperature and precipitation, number of rainy, frosty and snowy days, road curvatures, and presence of ramps. The selections of considered variables from certain categories, or in general, are different in each study, based on the data availability or the significance. The effect of covariates could vary on a case by case basis, but previous literature has provided strong evidence that driver behaviour covariates and non-behaviour covariates both significantly influence the occurrence of crashes.

A wide variety of statistical approaches has been used and developed over the last few decades. The Poisson regression model is the most basic and usually serves as a starting point in crash frequency estimation. The biggest drawback of being unable to deal with over-dispersion data (the variance exceeds the mean) has been addressed by the Negative Binomial (NB) model (also known as the Poisson-Gamma regression). The NB model offers a basic structure for model development and has been extensively applied in both earlier and recent studies. The ability to account for zero count observations in the above-mentioned models is usually not sufficient to handle predominant zeros in practice (regions where no crash occurred during the observation period). The zero inflated Poisson and NB model are developed for this circumstance, which has a splitting mechanism that provides two types of zeros governed by logit and probit distributions [19,20]. Although they are popular in traffic safety analysis, they have been criticised in highway safety for being unable to properly reflect the process of the crash-count generation due to the long-term zero means [21].

One methodological challenge in estimation is the existence of spatial unobserved heterogeneity and spatial and temporal correlation (neighbouring zones may share unobserved effects). These phenomena can be addressed with random-effects (in case of panel data), hierarchical and random parameters models. For the random-effects model, it is assumed that the effects remain constant over the spatial or temporal unit and follow certain distributions [22,23]. Such effects are also assumed to be uncorrelated with the considered covariates. The random parameters model, as an extension of the random effects model, allows the parameter of covariates to vary across observations. It has been applied to crash count estimation in previous literature [24,25], and has often showed a significantly better statistical fit than the traditional fixed parameters models. Hierarchical models are applied to analyse data grouped into clusters at one or more levels, and crash data can be regarded as an example. The main assumption in this type of model is that there may be a correlation among crashes with the same category of vehicle and location, which may be due to unobserved features related to vehicle type or location. It has been applied in a number of crash modelling studies [26,27].

Bivariate/multivariate models are necessary tools to analyse specific types of crashes such as crashes that lead to death or injury. These specific types of crash counts are not independent and can be solved by joint modelling, in which the correlation among the severity levels for each roadway entity has been explicitly taken into account [28,29]. The finite mixture model is also popular in examining heterogeneous populations. It was developed based on the assumption that unobserved groups, also named latent class, exist within the overall populations. The considerable potential offered by finite mixture models can provide important new insights for crash data analysis, and has been applied in many studies [30,31]. Other methods such as bivariate/multivariate, neural networks, support vector machine, etc. are also widely used for crash frequency prediction [32].

### 2.2. Crash Duration Methods

Crash duration typically consists of the following phases: detection, response, clearance, and recovery. The clearance phase has the most potential to reduce overall duration and is immediately affected by response agencies. This includes clearing the driveway of stuck cars, wrecks and debris, assisting stranded motorists, and assisting with traffic control and traffic management. Researchers have used various methods such as regression [33,34,35], hazard-based duration models [6,36], fuzzy system [37], classification tree [38], neural networks [39], Bayesian models [40], support vector machine [41], and hybrid models [42] to evaluate crash duration. 

In early studies, a Kolmogorov-Smirnov test was performed to determine the most appropriate distribution of the accident duration, and a lognormal distribution was found to provide the best fit [43,44]. Although hazard-based models are gaining popularity in duration analysis, there have been many improvements in models using regression and statistical tests in the past decade. Hou et al. (2013) [45] proposed a new probability model for response time, which is based on crash operational mechanism instead of empirical observations. Seven influential factors (disabled vehicles, debris, shoulder/median involved, fully enclosed, injuries involved, heavy truck involved, and weekends) were determined to be associated with longer response preparation delays. At the same time, eleven factors were found to have the opposite effect (abandoned vehicles, collisions, all lanes blocked, fires involved, work areas involved, AM peak, PM peak, summer, winter, high-occupancy vehicle lane accidents, and average daily traffic volume).

Researchers generally believe that if timely incident response measures are taken, traffic incidents can be quickly cleared [45,46]. However, Ding et al. (2015) [47] suggested that, due to similar characteristics and relatively simple processes, incidents with shorter response times may have higher priority. To reduce such self-selection bias and better understand the interaction between endogenous and exogenous variables on incident clearance times, they applied the endogenous switching model. They found only one variable (single lane blocked) associated with shorter clearance time and six factors were closely related with longer clearance time: all travel lanes blocked, total closure, injury involved, fire involved, heavy truck involved and traffic control. The authors of [48] explored the potential of a modularity-optimising community detection algorithm and association rule learning algorithm for large dimensional datasets. 

The hazard-based duration modelling method is appropriate for handling positive, censored, and time-varying duration data [49]. Its main superiority is the ability to capture time dependency, not just the duration alone, which allows explicit research on how various explanatory variables affect the conditional likelihood of incident clearance over time [50]. In the family of hazard-based models, accelerated failure time models (AFT) and proportional hazard models (PH) are two optional parametric methods that incorporate the effects of external independent variables on the hazard function. Whereas the effect of PH modelling is to multiply the hazard by a certain constant, an AFT assumes that the survival time is accelerated or decelerated by a constant factor, which captures the direct impact of exposure on survival time. The most representative studies for incident duration modelling using the family of hazard-based models are presented below.

In an early study, ref. [51] used an accelerated lifetime method underlying hazard function to analyse crash duration and frequency data. They claimed that log-logistic distribution can replicate duration data better than lognormal distribution. In addition, shorter response and clearance times were observed for crashes that occurred during peak hours and daytime; special events, motorist and vehicle characteristics were also found to impact duration. The study by [46] evaluated duration data using the proportional hazard-based model with multiple parametric distributions. This study indicated that the Weibull distribution with gamma heterogeneity was the best fitting model for incident detection and response duration, while the log-logistic distribution provided the best likelihood ratio statistics. In terms of modelling result, longer response times were observed during peak hours, nights, weekends, and when spillage or non-single occupancy vehicles were involved. 

A research study by [50] applied a hazard-based model to identify the variables that significantly affect the incident clearance time. This study also compared five common distributions underlying hazard function (exponential, Weibull, Lognormal, Log-logistic, Gamma) and the generalised F distribution, where each of these five distributions is a special form. The result clearly showed that the generalised F distribution provides a better fit for the incident clearance data than other parametric models within any reasonable confidence interval. Furthermore, the effects of incident characteristics are significantly different among different approaches. Regarding the estimate results, a shorter incident clearance time was observed at lower traffic volume, while a longer time was required during the winter. The authors [52] examined the effect of abandoned and disabled vehicles on freeway incident duration, in addition to detection mechanisms, towing times and various environmental and temporal factors. AFT hazard-based models with log-logistic distribution were applied for disabled vehicles, abandoned vehicles and combined models. The modelling results suggested that more lane segments and notifications through the HELP patrol program are associated with shorter clearance times, and shoulder involvement and inverse weather decreased disabled vehicle incident duration but increased correspondingly the duration of an abandoned vehicle incident. Besides, incidents involved with multiple vehicles and forced multiple lanes closed are associated with a shorter duration, as expected.

Apart from the parametric accelerated failure time model underlying hazard-function, some studies have applied semi-parametric or non-parametric models to analyse incident duration. Unlike fully parametric proportional models, the most commonly applied semiparametric model, the Cox regression model, uses a partial likelihood estimation approach to evaluate how explanatory variables alter the baseline hazard to avoid specific duration distribution assumptions [53]. Hou et al. (2013) [45] used this approach and found that injury involved, fire involved, summer, and mean annual daily traffic volume were related to time-constant effects; seven factors including single/multiple lanes blocked, short/medium/long response time, disabled vehicles and collision were associated with time-increasing factor-affected hazards; the rest (debris, abandoned vehicles, heavy truck involved, night, weekends, traffic control) were found to have the opposite effects. 

Zhang et al. (2014) [54] employed nonparametric regression based on the Kaplan-Meyer model to calculate survival function and hazard function in order to separately describe the spatial-temporal distribution features of the multi-influencing factors. Then they applied the COX proportional hazard model to analyse the co-evolution mechanism among them. The probability of the occurrence of traffic incidents was higher under a relatively large amount of traffic and most of the incidents were observed at non-bottleneck segments or at low running speed. In addition, the vast majority were minor collision between two cars, or one-car breakdowns.

As may be noticed from the above review, all studies on crash duration are conducted at a micro-level, i.e., considering each crash record separately. Therefore, almost all the covariates considered are location-specific. On the other hand, for crash frequency analysis, crashes are aggregated at a region-level, and the region-specific covariates are typically considered. To reconcile this limitation, we perform a macro-level analysis of both frequency and duration to determine the impact of region-level covariates.

## 3. Methodology

### 3.1. Crash Duration

The hazard-based model’s ability to consider time dependency allows researchers to examine how the conditional likelihood of incident clearance is affected by different external variables over time. To characterise duration data, the hazard-based approach uses two main formulations: hazard and survival functions. The survival function, commonly abbreviated as *S*(*t*), denotes the likelihood that an event will not occur by a given time t. The probability that the event durations will end at time *t*, assuming that the individual has not arrived at time *t*, is known as the hazard function, or *h*(*t*).

Two different ways of modelling incident duration data are the proportional hazard model (PH) and the accelerated failure time model (AFT) [55]. The PH model posits that the influential factors multiply the hazard function. A breach of this hypothesis may lead to inaccurate estimations. Therefore, the AFT model can be used as an alternative when the survival data does not meet the requirements of the PH model. The AFT model assumes that the effect of an explanatory variable can accelerate or decelerate the survival time by a constant. AFT is a linear model of the logarithm of event duration, making it easy to interpret [56]. The AFT model’s underlying distribution could be a log-normal, exponential, log-logistic, or Weibull distribution. Only the exponential and Weibull distributions can fit the PH model’s hypothesis. Thus, to better fit incident data, the AFT technique is used in this study [6]. 

Incident duration *T* is a continuous random variable with a cumulative distribution function *F*(*t*), also denoted as the failure function. This function gives the probability that the incident has occurred before duration *t*.

(1) F(t)=Pr(T≤t)

The survival function provides the probability that the duration of a traffic incident is greater than or equal to time *t*.

(2) S(t)=Pr(T≥t)=1−Pr(T≤t)=1−F(t)

The probability function *f*(*t*) can be expressed as

(3) $$f\left(t\right)=\frac{dF\left(t\right)}{dt}\;$$

The hazard function *h*(*t*) describes the instantaneous potential unit time of the incident, given that the individual has not ended up at time *t*. The slope of the hazard function can capture the duration dependence. A positive duration dependence is observed when the slope is positive, suggesting that a longer duration of the incident is associated with a higher possibility of the incident ending soon. The clearance of the incident is independent of the time when the slope is zero.

(4) $$h\left(t\right)=\frac{f\left(t\right)}{1-F\left(t\right)}=\frac{f\left(t\right)}{S\left(t\right)}\;$$

The AFT model describes how explanatory variables affect survival time rescaling. It is assumed that the vector of variables *X* and the log of survival time *T* are linear.

(5) ln(T)=βX+ε 

where *β* is the estimated parameter vector, and *ε* is the error.

The hazard rate is written as

(6) $$h\left(t,X\right)=\psi h_{0}\left(\psi t\right)$$

where *h*_0_(*·*) is the baseline hazard function, $\psi =\exp\left(-(\beta_{0}^{\prime }+\beta_{1}^{\prime }x_{1}+\beta_{2}^{\prime }x_{2}+\cdot \cdot \cdot +\beta_{n}^{\prime }x_{n}\right)=\exp\left(-\left(\beta^{\prime }X\right)\right)$, and *n* is the number of external variables.

### 3.2. Crash Frequency

Negative Binomial (NB) and Poisson models are the two most commonly utilised approaches to model count data, which are discrete and non-negative [57]. The Poisson regression model assumes that the mean of the incident count strictly equals its variance, i.e., $E\left(y_{i}\right)=Var\left(y_{i}\right).$ However, this equality does not hold in most practical cases of crash modelling, where the variance exceeds the mean, so the data is considered to be over-dispersed. Previous literature suggests that the NB model is preferred to Poisson due to its capability to address this issue [32,55]. Following the most common log-linear relationship, NB relaxes this assumption by adding an error term $\varepsilon_{i}$ to the mean of the Poisson model:

(7) $$\lambda_{i}=\exp\left(\beta X_{i}+\varepsilon_{i}\right)$$

where $\lambda_{i}$ is the Poisson parameter, which is also the expected incident frequency at location i. $X_{i}$ is the vector of given explanatory variables for location i, and β is the corresponding vector of estimated parameters. $\exp\left(\varepsilon_{i}\right)$ is gamma distributed with mean one and variance α. Therefore, the variance can be defined as $\lambda_{i}\left(1+\alpha \lambda_{i}\right)$, which is different from the mean. 

### 3.3. Accounting for Unobserved Heterogeneity

The implicit assumption for the above two statistical methods is that the estimated coefficients of any individual covariate are constant across observations—such specification is known as a “fixed-parameter (FP) approach”. It also assumes that vector X’s external variables capture all variations in incident duration and frequency. However, when the influence of explanatory variables is not completely homogeneous throughout observations, this assumption may be violated. Alternatively stated, there may be unobserved heterogeneity among incident duration and frequency in regions that are not adjacent or neighbours [31,58]. 

Statistical approaches such as random parameters models [31,59], latent class (finite mixture) models [30], a combination of latent class and random parameters models [60], and Markov-switching models [61] are widely used to address the problem of unobserved heterogeneity. In the current study, Latent Class models are estimated to account for unobserved heterogeneity in crash frequency and duration prediction. The latent class approach is a discrete finite mixture model to capturing unobserved heterogeneity. The observations are divided into Q latent classes, with the calculated coefficients of the variables treated as constants. Because it does not enforce a distributional assumption on coefficients, LCM is considered semi-parametric [31,58]. The incident duration *Y* can be written as

(8) $$Y=\beta_{q}X+\varepsilon_{q}$$

where $\beta_{q}$ is an unknown parameter vector for class *q* (*q* = 1, …, *Q*). $\varepsilon_{q}$ follows a normal distribution with a scale parameter $\sigma_{q}$. The Akaike and Bayesian information criteria values are used to determine the number of classes [62]. 

Random parameter models are more time intensive than fixed parameter models because the analyst must make parametric assumptions about the distribution of heterogeneity across observations. Choosing the number of classes and covariates in a class membership component is likewise difficult in latent class models, although relatively straightforward.

In summary, this study estimates Fixed Parameters Negative Binomial (FPNB) and Latent Class Negative Binomial (LCNB) models to evaluate crash frequency. Further, this study estimates Fixed Parameters Accelerated Failure Time (FPAFT) and Latent Class Accelerated Failure Time (LCAFT) models to evaluate crash duration.

### 3.4. Study Area and Data Collection

In this paper, the dataset, collected over 4.5 years, from January 2012 to June 2016, is used, accounted for 95,568 records of crashes. It covers the entire Sydney Metropolitan Area in New South Wales, Australia [63]. It provides comprehensive information about each incident, including the location, incident time, duration, number of lanes affected, incident detection mechanism, and a brief description of each incidence. It was found that crash duration is highly skewed (positively skewed in this dataset), which is consistent with the unique feature of survival time data. Extreme outliers can last for days and are possible because of accidents that required special response (e.g., chemical spill) or caused damages to infrastructure. Given the capability of the median in describing the central tendency of the data, the median crash duration of each zone within the observation period will be used as the dependent variable for duration analysis. There are approximately 0.6% of crash records that lack duration and 0.1% that lack spatial coordinates. These data were omitted from the final analysis. 

**Figure 1:**
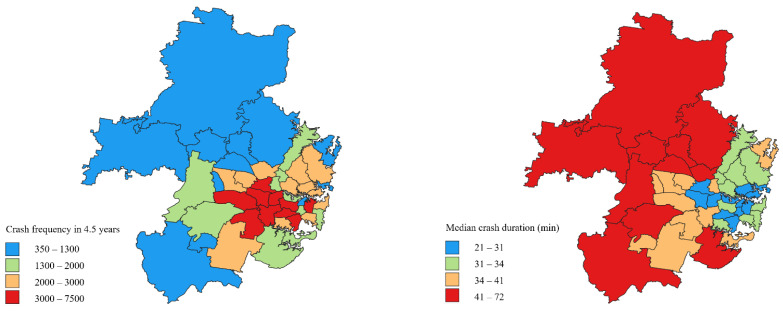
Crash frequency and the median crash duration of SA3. The zonal shapefile of SA3 was downloaded from the Australian Bureau of Statistics [64]. The original shapefile covered the entire state of New South Wales in Australia. Therefore, we trimmed the shapefile to restrict it to our study area, i.e., Sydney. Then we visualise our processed data (crash frequency and duration) spatially.

This study used the Statistical Area Level 3 (SA3) as the spatial aggregation to model crash duration and identify the contributing factors [64]. The crash frequency and median crash duration of these SA3s are presented in Figure 1. 

### 3.5. Independent Variables

Most of the independent variables for the current study are sourced from [65], which evaluated the impacts of zonal-level explanatory variables on the duration of vehicle breakdowns. A detailed description of the variables and the descriptive statistics can be found there. The explanatory variables included SA3-level socioeconomic characteristics, demographics, road network structure, etc. Apart from these variables, the current study also collects additional data in the form of the responder data obtained from websites of multiple government departments (Police Force, Ambulance, Health and Emergency Service) and the Australian Business Register Lookup (roadside assistance service) [66]. The descriptive statistics for the additional variables are provided in Table 1.

## 4. Results

### 4.1. Factor Analysis

In this study, 42 candidate explanatory variables were examined, which included 11 which were road network structure related and 31 others. To overcome multicollinearity, a linear dimension reduction approach is used. The principal component factor analysis method is employed [67], and a varimax rotation with Kaiser Normalization is conducted to define probabilistic loadings. The number of factors depends on when the eigenvalues displayed by all factors are greater than one. Only the loadings (the Pearson correlation between the variables and the extracted component) greater than 0.4 are retained for interpretative purposes, as suggested by [68]. If the loading value is higher, it shows that the variable is strongly correlated to the factor that it belongs to. In addition, the direction of correlation is indicated by the sign of the loading. Eight factors had eigenvalues greater than one, accounting for 84% of the variance. The results of the factor analysis are shown in Table 2. The study by [65] performed two separate factor analyses for network structure and other variables, whereas in this study, the factor analysis is performed once for all the variables combined. The factors are discussed in length in the “Discussion” section.

The factors are labelled according to the underlying variables that have a strong influence on the factor. For example, the first factor is termed “Inexperience and Unaffluent” because of the strong loading of variables such as driving licenses and income. It has been named thus, instead of “Income and Affluent”, due to the negative factor loading of the variables. This labelling makes it simple to interpret the modelling results. 

The second factor, Land use homogeneity, is mainly composed of fuel utilisation and land use. In terms of land use, relatively remote areas are expected to have more diversified land use. In addition to the commercial and residential areas that each region is equipped with, the proportion of undeveloped land and land used for industrial, agricultural, and shipping purposes is higher in relatively remote areas. Road cargo transportation is considered as a crucial factor in adding value and improving productivity in these industries, so it is reasonable that more heavy vehicles are registered in these SA3s. Furthermore, most heavy vehicles operate on diesel. Besides, the trips used for shipping purposes are usually long-distance, high speed and intercity, and are generally made on motorways and freeways in relatively remote areas. Such a hypothesis explains the correlation between road length and the second factor. 

The third component, Density, primarily consists of population and road network densities. In low-density areas, the public transportation system may not be well-developed or widely covered, and more people may need cars to meet their travel needs. The fourth factor is mainly comprised of the amount of response service depots within an SA3. The subsequent factors, Connectivity and Hierarchy [70], are two fundamental network structure characteristics that are highly correlated with their corresponding indicators as expected. The seventh component, the Exposure factor, is widely used to measure vehicle usage. Finally, the eighth component describes public transport usage. 

### 4.2. Modelling Results

We used the Anderson-Rubin approach to determine the factor scores for each zone (SA3) after identifying the factors [71]. In the crash frequency and duration models, we used these scores as the explanatory variables. For the duration model, various model specifications were fitted by changing the distribution of the hazard function. The results of both the FP and LC approaches are shown in the paper to demonstrate the better model fit of the LC approach.

Table 3 and Table 4 display the modelling results for crash duration and frequency, respectively. In terms of duration analysis results, the final comparison only shows the best hazard distribution inside the FPAFT (log-logistic) and LCAFT (Weibull) models. The negative sign of the estimated parameter suggests that an increase in the factor is associated with a decrease in the crash duration. The best-fit LCAFT model has two latent classes, with Class-1 including 37% of SA3s and Class-2 including 63% of SA3s. Further, the LC-AFT model with Weibull distribution is preferred, and performed better than the FP model. 

As described in Table 4, FPNB and LCNB models were fitted to crash frequency data. A positive sign of an estimated coefficient suggests an increase in crash frequency associated with an increase in that factor and vice versa. The best-fit LCNB model has two classes, showing that unobserved heterogeneity is present in the crash data. The following section offers some intriguing insights using statistical modelling. 

## 5. Discussion

In explaining the impact of significant factors, crash duration and frequency discussion are restricted to the best-fitting model, the LC-AFT model, with Weibull hazard distribution and the LC-NB model, respectively. 

### 5.1. Inexperience and Unaffluent

The majority of SA3s, i.e., those belonging to Class-2, observe an increasing crash duration with decreased income and experience, whereas those belonging to Class-1 show the opposite relation. In minor crashes, more experienced drivers (no license restrictions) are likely to be more proficient, resulting in faster reporting and reaction times. Because higher-income persons have more work-related obligations, immediately calling the police and/or notifying insurance representatives may be the best solution. Furthermore, the SA3s with higher income are correlated with newer vehicles (i.e., aged less than five years), as seen in Table 2. Newer vehicles are superior in terms of safety. Besides, safety standards and regulations have developed over the years, providing more affordable vehicles with the latest safety features [72]. Therefore, the severity and casualty of crashes with newer vehicles tend to be lower, resulting in a shorter clearance duration.

While the factor has contrasting impacts on duration in the two latent classes, it has a consistent (same direction of impact) impact on crash frequency. The LCNB model indicates that, as experience and income increase, the number of crashes decreases, which is consistent with past studies [73]. Mechanical failure occurring during driving is one of the leading non-artificial reasons causing crashes. Newer and more high-end vehicles usually have better safety performance. The willingness and financial capability of higher-income earners to purchase these vehicles may be higher. According to [74], the two most common issues that lead to crashes are tires and wheel-related problems and brake failure, and worn-out components are the main contributing factor. Under the same maintenance frequency, vehicles aged more than ten years old will bear a higher risk of crashes caused by mechanical failure than newer vehicles in general. 

### 5.2. Land Use Homogeneity 

It is observed that the increase in land use homogeneity reduces the crash duration in both latent classes. In heavy vehicle crashes, response services are limited compared to light vehicle crashes, causing a lengthier response time. Towing, roadside diagnostics, and repair may also take longer. Heavy vehicles also usually transport cargo or passengers. Therefore, the response efficiency may depend significantly on the availability of rescue resources (e.g., ambulance, trailer and heavy load crane). Usually, post-incident management takes a longer time compared to light vehicles. Responders may encounter more intersections in regions with shorter average road lengths, causing a delay in arriving at the incident location. The above hypothesis can also be applied to the Density Factor.

The increase in this factor increased the crash frequency. The compositions of diesel are not as active or volatile as petrol, and they are less likely to form explosive mixtures. Therefore, diesel-powered vehicles are less likely to be accidentally ignited or exploded. In addition, diesel engines usually have lower speeds, resulting in slower aging and wear than petrol engines. These vehicles are equipped with fewer auxiliary appliances and do not require an ignition system [75]. As a consequence, diesel vehicles may contribute less to crashes caused by component failures. 

These possible reasons could also be applied to heavy vehicles. Besides, due to the low manoeuvrability and blind spots of heavy vehicles, drivers of light vehicles tend to be more cautious and patient when approaching them.

### 5.3. Density Factor

The results suggest that, as the density factor increases, crash duration decreases but crash frequency increases. Regions with higher network density could cause more traffic conflicts because of multiple crossing movements and approaches [12,17,76]. A report from [77] indicates that approximately 40% of crashes occurred at intersections. Higher network density is also a proxy for traffic congestion, as frequent encountering of intersections would lead to a reduction in traffic speeds. Thus, although there are more conflicts (and crashes), their severity (and consequently duration) may not be high [78].

As the number of families with children in SA3s increases, duration increases but frequency decreases. The reduction in crash frequency is likely to be because of the extra awareness and protection by parent drivers [57]. On the other hand, the increase in duration could be attributed to children’s special medical care requirements. The anatomy and physiology of children differ significantly from those of adults. Therefore, when crashes happen, the impact on the head is more prominent in children than adults. For example, serious head injuries are seen in 80% of children who die from multiple organ injuries, but the figure is 50% in adults [79].

### 5.4. Responder Factor

An increase in the Responder factor decreases the crash duration in 63% of SA3s, i.e., those belonging to Class-2. However, the factor is insignificant for Class-1. High availability of responders may help shorten response and clearance time and minimise the possibility of casualties caused by rescue or medical care delays. It is also worth mentioning that both endogeneity bias and self-selection are observed in incident duration phases [47]. Higher priority is usually granted when a severe fire or fatality incident occurs, resulting in a faster response. This faster response could effectively prevent the crash from becoming more severe, leading to a shorter clearance duration.

However, the responder factor indicated an increased crash frequency in both the latent classes of the LCNB model. The availability of responders does not contribute directly to crash frequency. A possible explanation for the result is that previous incident records may have been used in setting up more responders. This hypothesis may generate an endogeneity problem, and further study is required [80]. 

### 5.5. Connectivity Factor

The increase in road connectivity increases crash duration in the minority of SA3s (class-1). Strongly connected networks feature more links connecting each node, indicating greater complexity and a longer signal cycle (in the case of signalised intersections). Delays at such intersections may result in lengthier reaction times.

In Class-2 of the LCNB model, well-connected networks are expected to have fewer accidents. A network with poor connectivity is usually associated with more dead ends and loop streets. Compared with the traditional grid pattern, this network layout may restrict motorists’ sight distance, resulting in decreased perception, longer reaction time and greater difficulty in reducing vehicle speed [81]. All of these impacts might increase the risk of a crash for vulnerable road users in poorly connected networks. The authors of [82] mentioned that suburban areas with high connectivity could effectively distribute local traffic by collector roads parallel to the arterials. Therefore, the safety of arterials could be improved thanks to decreasing local traffic access. Highly connected networks are usually associated with more complex intersections such as roundabouts and signalised intersections. These have usually been adequately designed and assessed by transportation authorities to ensure the safety of various road users crossing from different directions [77]. The other contribution may be that highly connected networks retain the capacity to dissipate the congestion, which could lower the need to build more arterials and generate less travel in a city-wide network [11]. 

### 5.6. Hierarchy Factor

In both classes, crash duration decreases with the presence of more high-functional roads. This is likely to be due to better detection infrastructure and faster identification of problems on higher functional class edges [83]. Higher functional class edges are usually built with more lanes. Therefore, when involved in incidents, the possibility of a complete road blockage is relatively lower than for lower functional class edges. Under this circumstance, the delay due to congestion could be reduced.

Networks with a higher hierarchy are associated with more crashes, as indicated by the positive coefficient in the LCNB model. Higher functional class edges are usually built with more lanes and are expected to have higher traffic volume. On the one hand, it could be explained by the increasing number of sudden lane-changing related crashes, accounting for approximately 17% of severe accidents [84]. On the other hand, various pieces of literature have identified a positive correlation between traffic volume and accident frequency [85,86]. In addition, excessive speed driving could be more frequent on roads with a higher speed limit, which also contributes to higher crash frequency. Finally, the hierarchy may indirectly increase the exposure of vehicles by encouraging auto dependency and generating unnecessary travel distance. 

### 5.7. Exposure Factor

A negative coefficient of the exposure factor was observed in class-1 of the LCAFT model. Higher exposure in a region usually indicates higher traffic volume in general, which could induce more congestion and lower the average vehicle speed of responders. Acknowledging that this is also associated with more crashes, the availability of responders may decrease, and their average travel distance may increase. Both hypotheses are responsible for longer response duration. However, minor incidents may allow drivers to gain expertise and reduce time. Crash frequency increases as the level of vehicle exposure increases. This is explained by increased driving time and vehicle wear and tear.

### 5.8. Public Transport Proportion Factor

A negative impact was observed on crash duration, and the opposite effect was observed on frequency. Traffic congestion in urban areas continues to grow; the use of public transportation will also increase simultaneously to meet people’s daily travel needs. The factor analysis shows a negative association between public transportation and car proportion variables, and the reasoning for the Density Factor may apply here as well.

### 5.9. General Discussion

As presented in Figure 1, zones with higher crash frequency are generally associated with a shorter duration, most likely due to the proportion of minor crashes. The overall trend also basically matches the distribution of remote areas and areas that are densely populated and developed in Greater Sydney. The majority of factors are observed to show different sides of impacts on crash duration and frequency (i.e., increase in duration but a decrease in frequency as a factor increases and vice-versa). Besides, many underlying explanatory variables are living conditions and lifestyle measures, urban quality, and incident management resources, which are unevenly distributed in these two categories of region. Due to higher vehicle exposure, the crash frequency in densely populated and urban environments is expected to be much higher. These areas are usually associated with higher traffic congestion and lower vehicle speeds, which can reduce the proportions of fatalities and severe injuries. In this case, thanks to the reduced difficulty and complexity of the response, the overall crash duration can be shortened. It is also worth noting that the significance and scale of the effect are quite different in these areas. For example, a minor crash closer to the downtown area may severely affect the traffic flow of up to several kilometres of roadway. As a result, the response team may be blocked, and the risk of secondary crashes may increase. On the other hand, uneven service demand (incident occurrence) may lead to uneven distribution of response resources. In regions that experience the longest duration, it is expected that additional time required will be spent on the preparation and travel of the response team. Lastly, the discrepancy in travel patterns and driving behaviours is also a contributor. These variables have not been adequately considered in this study and could generate diverse or even opposite effects. Therefore, a comprehensive understanding of crash frequency and duration based on the same dataset will help develop optimal countermeasures and post-incident management plans.

## 6. Conclusions

Past research efforts have been placed on analysing vehicle crashes with respect to frequency, risk, severity, and duration. Regarding crash duration, a branch of studies is devoted to developing real-time duration prediction models that traffic operation centres could employ. The existing studies concentrated on micro-level or localised explanatory variables. These variables include the time of day, weather, distance from the city centre, road geometry, posted speed, type and number of vehicles involved, etc. On the other hand, the effects of zonal factors such as road network features, demographic characteristics, environmental properties, etc., have not been thoroughly examined, contrary to studies on frequency. Hence, fully understanding how macro-level factors affect incidents and subsequent management processes can help develop the most suitable strategies for different regions.

With this intention, the current study described the modelling of vehicle crash duration and frequency data from the Greater Sydney Metropolitan Area. After a preliminary dimensionality reduction process, a total of eight factors in terms of socioeconomic, vehicle utilisation, environmental, responder, and network structure features were examined as candidate variables. Factor scores were computed, and different statistical models were developed to evaluate crash frequency and duration.

With regard to modelling results, the LCAFT model with Weibull distribution and the LCNB model were the most suitable for crash duration and frequency, respectively. This study’s findings are interesting for congestion management and reduction, especially in metropolitan settings. Network administrators and multiple response service personnel can implement the findings to develop more efficient incident clearance strategies and distribute response resources more reasonably. Results also proved that network density and structure are associated with the number of crash occurrences. In transportation safety studies, taking network structure characteristics into account may aid in the planning of safer and more sustainable communities. Even though the overall structure of the road network has already been decided in some locations, the results can still be used by the relevant authorities to reduce traffic congestion and the danger of secondary events (e.g., ideal position of emergency service depot, emergency lane, and fast detection devices).

A key limitation of this study is the lack of sufficient sample size. Another limitation is the lack of consideration of temporal stability and transferability in our considered time range (i.e., from January 2012 to June 2016). This could be an issue because the data related to various explanatory variables are collected at different time intervals. The study is only employed the most fundamental road network measures. Nevertheless, any additional data in terms of street-level data (e.g., average shoulder width and the proportion of road length with curbs) and road network measures such as network pattern may increase the model’s usefulness.

Subsequent research can be conducted in order to account for more street network measures such as circuity and network pattern. The crash frequency and median duration of SA3 were applied in the current study as dependent variables for the statistical model development. The temporal stability of dependent variables can also be jointly modelled and used to validate current results. Further research can be applied to other geographical regions and by including more regions in the analysis.

## Figures and Tables

**Table 1:** Descriptive statistics of the variables.

Variable	Mean	SD	Minimum	Maximum
Median vehicle crash duration (minutes)	37.15	9.06	21	72
Crash frequency	2171	1420.51	350	7159
**Independent variables (apart from the ones already considered in [65])**				
Total number of roadside assistance services	7.75	7.93	0	36
Total number of emergency services	0.93	1.26	0	6
Total number of police stations	2.50	2.33	0	11
Total number of ambulance stations	1.25	1.10	0	4
Total number of public hospitals	1.32	1.46	0	6

**Table 2:** Rotated factor loading of all candidate variables.

Candidate Variables	Component							
	1—Inexperience and Unaffluent	2—Land Use Homogeneity	3—Density	4—Responder	5—Connectivity	6—Hierarchy	7—Exposure	8—Public Transport Proportion
Proportion of P2 license holders	0.937							
Proportion of P1 license holders	0.931							
Proportion of unrestricted license holders	−0.931							
Proportion of income earners	−0.916							
Average yearly income of income earner (in $10,000)	−0.863							
Proportion of white-collared workers among total employees	−0.835							
Proportion of LPG vehicles	0.834							
Proportion of learners’ license holders	0.798							
Proportion of vehicles older than 10 years	0.729							
Proportion of vehicles aged between 5 to 10 years	−0.724							
Proportion of people who speak a language other than English at home	0.649							
Total number of roadside assistance services	0.607			0.523				
Average precipitation per day (in mm)	−0.591	0.441						
Proportion of vehicles aged less than 5 years	−0.562					0.422		
Pproportion of petrol powered vehicles		0.927						
Proportion of diesel powered vehicles		−0.908						
Land use entropy		−0.823						
Proportion of heavy vehicles	0.515	−0.734						
Road length (km)		−0.670						
Proportion of individuals born overseas		0.483	0.472					
Average daily temperature (°C)		0.481	0.429					
Entropy of length-weighted spatial orientation of roads [69]		−0.460						
Intersection density (intersections/sq.km.)			0.920					
Road density (km/sq.km.)		0.402	0.886					
Population density		0.404	0.826					
Rratio of car users to other mode users			−0.738					
Proportion of families with children under 15 years			−0.698					
Number of speed cameras				0.788				
Total number of emergency services				0.787				
Total number of police stations				0.783				
Total number of ambulance stations				0.668				
Total number of public hospitals			0.421	0.646				
Meshedness coefficient					0.876			
Link-node ratio (# links/# intersections)					0.873			
Average node degree					0.713			
Proportion of Motorway, trunk and primary roads (MTP)						0.850		
Average number of lanes						0.693		
Entropy of length-weighted road type						0.669		
Average daily weighted travel distance (in million-km)							0.753	
Total number of registered vehicles (in 10,000)							0.707	
The ratio of public transport users to other mode users			0.500					0.522

**Table 3:** Crash duration model estimation results.

	FPAFT Model—Logistic		LCAFT Model—Weibull			
			Class-1		Class-2	
	B	*p*-Value	B	*p*-Value	B	*p*-Value
Constant	3.59	<0.01	3.65	<0.01	3.60	<0.01
Inexperience and Unaffluent	-	-	−0.02	<0.01	0.04	0.02
Land use homogeneity	−0.13	<0.01	−0.14	<0.01	−0.12	<0.01
Density	−0.10	<0.01	−0.11	<0.01	−0.13	<0.01
Responder	-	-	-	-	−0.06	0.05
Connectivity	-	-	0.03	<0.01	-	-
Hierarchy	−0.08	<0.01	−0.03	<0.01	−0.09	<0.01
Exposure	-	-	−0.06	<0.01	0.02	0.10
Public transport proportion	−0.04	0.02	−0.12	<0.01	-	-
Sigma (Scale parameter)	0.06	<0.01	0.01	<0.01	0.06	<0.01
Class Probability	-	-	0.37	<0.01	0.63	<0.01
AIC	−64.1		−84.4			
BIC	−53.4		−47.0			
Loglikelihood	38.03		63.22			

**Table 4:** Crash frequency model estimation results.

	FPNB Model		LCNB Model			
			Class-1		Class-2	
	B	*p*-Value	B	*p*-Value	B	*p*-Value
Constant	7.49	<0.01	7.76	<0.01	7.45	<0.01
Inexperience and Unaffluent	0.24	<0.01	0.41	<0.01	0.14	<0.01
Land use homogeneity	0.3	<0.01	0.36	<0.01	0.27	<0.01
Density	0.3	<0.01	0.55	<0.01	0.27	<0.01
Responder	0.22	<0.01	0.47	<0.01	0.24	<0.01
Connectivity	-	-	-	-	−0.08	0.07
Hierarchy	0.28	<0.01	0.63	<0.01	0.27	<0.01
Exposure	0.28	<0.01	0.5	<0.01	0.21	<0.01
Public transport proportion	0.12	<0.01	-	-	0.16	<0.01
Alpha (Overdispersion parameter)	0.06	<0.01	-	-	82.24	0.03
Class Probability	-	-	0.32	<0.01	0.68	<0.01
AIC	677.3		624.5			
BIC	693.3		662			
Loglikelihood	−329.64		−291.27			

## Data Availability

The data presented in this study are available on request from the corresponding author.
